# Supplementary material for: Intracoronary-Cardiosphere-Derived Cell Secretome Therapy: Effects on Ventricular Tachycardia Inducibility and Cardiac Function in a Swine Model
Source: Biomedicines. 2025 Apr 25;13(5):1043. doi: 10.3390/biomedicines13051043 (PMC12108644; doi:10.3390/biomedicines13051043)
Supplement: Supplementary file 1 [file biomedicines-13-01043-s001.zip › biomedicines-3574864-supplementary.pdf]

Supplementary Material

Intracoronary cardiosphere derived cell secretome therapy: Effects on ventricular tachycardia inducibility and cardiac function in a swine model

Table S1. Evolution of blood-derived parameters and reference range for swine in our institution. Data presented as mean±standard deviation. No statistically significant differences were observed between groups in any blood derived parameter p>0.05). Intragroup comparisons revealed statistically significant differences (*p-value) in S-CDCs (GOT, GPT, CRP, Total Proteins and Troponin I) and in CON (GOT, CRP, Total Proteins, Urea, Troponin I and CK-MB), although values remained within clinically acceptable limits during the complete follow-up period.

Group	Reference Values	S-CDCs				CON			
Timepoint		Pre-therapy	Post-therapy	4 Months Post-therapy	p-value	Pre-therapy	Post-therapy	4 Months Post-therapy	p-value
Creatinin (mg/dl)	<2.30	2.23±0.30	2.35±0.31	2.17±0.63	0.61	2.65±0.29	2.65±0.31	2.38±0.41	0.12
Glucose (mg/dl)	<150	57.00±11.66	37.67±24.60	61.67±12.86	0.20	67.67±16.13	80.33±29.86	59.40±15.22	0.22
GOT (U/l)	<80	59.40±15.22	60.00±22.02	23.33±6.38	0.01*	20.67±9.50	52.00±19.82	23.80±7.22	0.01*
GPT (U/l)	<60	32.67±8.41	50.67±10.29	25.83±1.47	0.01*	30.50±12.65	40.83±5.95	25.00±9.59	0.07
CRP (mg/l)	<8	0.41±0.34	0.84±0.46	0.00±0.00	0.02*	0.39±0.41	0.83±0.39	0.00±0.00	0.01*
Total Proteins (g/dl)	<8.30	4.72±0.55	6.88±0.86	6.23±0.88	0.01*	4.97±1.04	6.13±0.47	6.37±0.60	0.03*
Urea (mg/dl)	<30	28.02±5.27	27.22±6.62	23.12±4.98	0.14	32.42±4.23	33.57±5.17	23.00±3.07	0.01*
Troponin I (µg/l)	<0.10	0.01±0.01	0.06±0.08	0.01±0.01	0.02*	0.02±0.01	0.09±0.10	0.00±0.00	0.01*
CK-MB (µg/l)	<12	4.02±1.51	4.30±2.09	3.88±3.06	0.25	5.98±4.15	6.87±4.61	2.34±0.51	0.02*

Table S2. Evaluation of macroscopic lesions and histology of the myocytes in the DS area. No significant differences between groups were observed in any parameter.

Macroscopic Lesions				
**Parameter**	**Finding**	**CON (n=7)**	**S-CDCs (n=7)**	**p-value**
**Edema**	**Absent**	14.3%	0%	**0.720**
	**Mild**	14.3%	42.9%	
	**Moderate**	57.1%	57.1%	
	**Severe**	14.3%	0%	
**Hemorrhage**	**Absent**	85.7%	71.4%	**0.382**
	**Mild**	14.3%	14.3%	
	**Moderate**	0%	14.3%	
	**Severe**	0%	0%	
**Necrosis**	**Absent**	100%	85.7%	**1.000**
	**Mild**	0%	0%	
	**Moderate**	0%	0%	
	**Severe**	0%	14.3%	
**Inflammation**	**Absent**	0%	0%	**1.000**
	**Mild**	100%	1000%	
	**Moderate**	0%	0%	
	**Severe**	0%	0%	
**Histology of the Myocytes**				
**Parameter**	**Finding**	**CON (n=7)**	**S-CDCs (n=7)**	**p-value**
**Hyperthrophy**	**Absent**	14.3%	0%	**1.000**
	**Present**	0%	0%	
	**Not Determined**	85.7%	100%	
**Myocytolysis/ Edema/ Vacuolar Degeneration**	**Absent**	0%	0%	**1.000**
	**Mild**	100%	100%	
	**Moderate**	0%	0%	
	**Severe**	0%	0%	

.
